# ASSOCIATIONS OF PAIN INTENSITY WITH SEDENTARY BEHAVIOR AND PHYSICAL ACTIVITY IN OLDER ADULTS

**DOI:** 10.1093/geroni/igz038.074

**Published:** 2019-11-08

**Authors:** Kushang Patel, Andrea LaCroix, Paul Crane, Rod L Walker, KatieRose Richmire, Eric B Larson, Dori E Rosenberg

**Affiliations:** 1 University of Washington, Seattle, Washington, United States; 2 University of California San Diego, La Jolla, California, United States; 3 Kaiser Permanente Washington Health Research Institute, Seattle, Washington, United States

## Abstract

Exercise is recommended for several painful, age-associated conditions; however, relationships between pain intensity and objectively measured sedentary behavior and physical activity have not been investigated in older adults. Accordingly, we analyzed cross-sectional data on 936 older adults in the ACT Study who self-reported their pain intensity on a 0-10 rating scale (0=no pain; 1-3=mild pain; and 4-10=moderate/severe pain) and wore an activPAL accelerometer. A total of 181 (19.3%) reported no pain, while 564 (60.3%) and 191 (20.4%) reported mild and moderate/severe pain, respectively. Linear regression models adjusted for age and sex estimated that compared to those with no pain, participants with moderate/severe pain walked significantly fewer steps/day (b-coefficient=-778 [95%CI: -1377, -179]) and had fewer sit-to-stand transitions/day (b-coefficient=-2.9 [95%CI: -5.6, -0.1]). In contrast, there were no significant differences in these outcomes comparing no pain versus mild pain. Future research will examine effects of pain treatments (opioids) and diagnoses on accelerometer-measured outcomes.

